# Environmental and topographic drivers of amphibian phylogenetic diversity and endemism in the Iberian Peninsula

**DOI:** 10.1002/ece3.9666

**Published:** 2023-01-06

**Authors:** Maria João Paúl, Dan Rosauer, Pedro Tarroso, Guillermo Velo‐Antón, Sílvia B. Carvalho

**Affiliations:** ^1^ CIBIO, Centro de Investigação em Biodiversidade e Recursos Genéticos, InBIO Laboratório Associado, Campus de Vairão Universidade do Porto Vairão Portugal; ^2^ BIOPOLIS Program in Genomics, Biodiversity and Land Planning CIBIO Vairão Portugal; ^3^ Departamento de Biologia, Faculdade de Ciências Universidade do Porto Porto Portugal; ^4^ Division of Ecology and Evolution, Research School of Biology and Centre for Biodiversity Analysis The Australian National University Canberra Australian Capital Territory Australia; ^5^ Departamento de Ecoloxía e Bioloxía Animal, Grupo de Ecoloxía Animal, Torre Cacti (Lab 97) Universidade de Vigo Vigo Spain

**Keywords:** diversity measures, evolutionary history, null models, spatial autoregressive models, spatial diversity patterns

## Abstract

Understanding the ecological and evolutionary processes driving biodiversity patterns and allowing their persistence is of utmost importance. Many hypotheses have been proposed to explain spatial diversity patterns, including water‐energy availability, habitat heterogeneity, and historical climatic refugia. The main goal of this study is to identify if general spatial drivers of species diversity patterns of phylogenetic diversity (PD) and phylogenetic endemism (PE) at the global scale are also predictive of PD and PE at regional scales, using Iberian amphibians as a case study. Our main hypothesis assumes that topography along with contemporary and historical climate are drivers of phylogenetic diversity and endemism, but that the strength of these predictors may be weaker at the regional scale than it tends to be at the global scale. We mapped spatial patterns of Iberian amphibians' phylogenetic diversity and endemism, using previously published phylogenetic and distribution data. Furthermore, we compiled spatial data on topographic and climatic variables related to the water‐energy availability, topography, and historical climatic instability hypotheses. To test our hypotheses, we used Spatial Autoregressive Models and selected the best model to explain diversity patterns based on Akaike Information Criterion. Our results show that, out of the variables tested in our study, water‐energy availability and historical climate instability are the most important drivers of amphibian diversity in Iberia. However, as predicted, the strength of these predictors in our case study is weaker than it tends to be at global scales. Thus, additional drivers should also be investigated and we suggest caution when interpreting these predictors as surrogates for different components of diversity.

## Graphical abstract

Our study addresses the drivers of biodiversity patterns. We build on existing research regarding spatial diversity patterns to identify potential drivers of species diversity patterns, using Iberian amphibians as a case study. Our findings show that water‐energy availability and historical climate instability are the most important drivers of diversity in our study. However, the strength of these predictors in our case study is weaker than it tends to be at global scales.

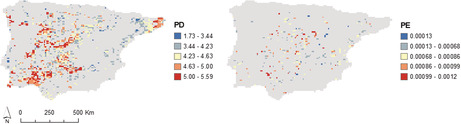

## 1 INTRODUCTION

Global biodiversity is declining at an exceptionally high and unprecedented rate (Butchart et al., 2010; Mace & Purvis, 2008; Pimm et al., 2014) that is likely to increase in the future (Mace & Purvis, 2008). This implies not only a loss of taxonomic diversity but also of evolutionary history and the ecosystem services provided by current biodiversity levels.

Biodiversity has several facets, including diversity within taxonomic groups, species, and of ecosystems, that work at different scales of space and time (Tucker & Cadotte, 2013). It has been argued that biodiversity measures should account for the evolutionary relationships between species (Vane‐Wright et al., 1991). Previous studies suggest that phylogenetic trees allow the prediction of phenotypic, genetic, and/or behavioral differences between evolutionary lineages (Niepoth & Bendesky, 2020; Rapacciuolo et al., 2019; Tucker et al., 2017). Thus, several metrics have been developed to measure different aspects of evolutionary history (Tucker et al., 2017). Such measures have been applied to describe spatial patterns of biodiversity (Fritz & Rahbek, 2012), to infer the ecological and geological processes that shaped those patterns (Davies & Buckley, 2011), and to identify priority areas for conservation optimized for the protection of evolutionary history (Carvalho et al., 2017; Gumbs et al., 2020; Pollock et al., 2017; Rosauer et al., 2018). Phylogenetic diversity (PD; Faith, 1992) is one of the most popular diversity measures. It is a measure of biodiversity richness accounting for the shared evolutionary history between taxa and is calculated as the sum of branch lengths of a phylogenetic tree for a given set of species (Tucker et al., 2017).

Centers of endemism are areas where components of biological diversity with restricted ranges are concentrated (Rosauer et al., 2009; Slatyer et al., 2007) mostly due to a unique combination of persistence of lineages, vicariance, and speciation in geographical isolation (Crisp et al., 2001). These areas are usually perceived as having high value for conservation (Daru et al., 2019; Rosauer et al., 2009; Rosauer & Jetz, 2015), although different taxonomic classifications, endemism metrics, and spatial resolution can affect their identification and effective protection (Daru et al., 2020; Shipley & McGuire, 2022). Phylogenetic endemism (PE; Rosauer et al., 2009) combines weighted endemism (WE; Crisp et al., 2001; Slatyer et al., 2007) and PD to identify areas where components of PD are spatially restricted. Therefore, PE is calculated as branch length divided by geographical range, summed for all branches linking a set of taxa through the root. In other words, the length of each branch is divided across all of its geographical ranges. Similarly to PD, this measure does not depend on taxonomic rank, making it more robust to taxonomic changes and inflation, i.e., when subspecies are raised to species due to changes in the species concept (Isaac et al., 2004). In a given location, PE depends on: (i) the geographical range of the taxa occurring there; (ii) how much evolutionary history is shared with closely related taxa, and (iii) the geographical range of those related taxa (Rosauer et al., 2009). High PE is found where long branches are restricted to a small range (Rosauer & Jetz, 2015).

Spatial patterns of biodiversity at different evolutionary scales are structured through an interplay of evolutionary processes and geological, ecological, and environmental changes (Wiens & Donoghue, 2004). Thus, conservation strategies are progressively focusing on preserving the processes that generate and maintain genetic diversity as well as adaptive potential, i.e., the capacity of a population or species to evolve in response to environmental change (Mace & Purvis, 2008; Moritz, 2002; Pressey et al., 2007). To properly address these evolutionary processes, it is necessary to understand the mechanisms that create and maintain phylogenetic endemism, as these are crucial to preserving biodiversity, identifying biodiversity proxies that can aid conservation planning and predicting possible impacts and responses to climate change (Araújo et al., 2008; Moritz & Agudo, 2013; Rosauer & Jetz, 2015).

Several of the topographic and environmental hypotheses that have been formulated to explain spatial patterns of phylogenetic endemism are mostly related to some kind of niche facilitation, given its general correlation with species richness (Murali et al., 2021; Rosauer & Jetz, 2015). Relevant hypotheses include the water‐energy availability, topography, and historical climatic stable refugia (Table 1). However, areas with high species richness or endemism are not always congruent with high PD or PE, and vary across different tetrapod classes (Murali et al., 2021) and spatial scales (Daru et al., 2020). Comprehensive tests on the drivers of spatial patterns of phylogenetic diversity have been more focused on global scales, whereas studies focusing on the processes that drive PE at regional scales have been more scarce (but see Barratt et al., 2017; Carnaval et al., 2014; Rosauer et al., 2015). Furthermore, no regional studies focused on the drivers of PE have included examples in the Western Palearctic.

**TABLE 1:** Hypothesized predictors of spatial diversity patterns.

Hypothesized drivers	Description	References
Water and energy availability	Water‐energy availability is expected to enhance long‐term survival by offering resources for conversion into food and, thus, supporting viable populations and species co‐existence.	Araújo et al. (2008); Evans et al. (2005); Hawkins et al. (2003)
Topography	Mountain ranges harbor high levels of diversity and endemism and also favor isolation processes via the barrier effect.	Antonelli et al. (2018); García‐París et al. (2000); Rahbek et al. (2019)
Historical refugia	Climate stability or refugia favor species´ survival and persistence in climatic refugia. Thus, climatic stable areas are expected to have higher diversity and endemism. Population isolation in these climatic refugia often results in differentiation and vicariant speciation processes.	Araújo et al. (2008); Carnaval et al., 2009; Gómez and Lunt (2007); Hewitt (1996); Wiens and Donoghue (2004)

The Iberian Peninsula (IP) is included in the Mediterranean biodiversity hotspot (Myers et al., 2000), holding several endemic species and many unique evolutionary lineages. Thus, it is a remarkable area to research contemporary and historical drivers of spatial distribution patterns of PE at a regional scale (Hewitt, 1996). Since the separation of the IP from the African continent after the Messinian Salinity Crisis (MSC) at the end of the Miocene (5.3 Mya), diversification processes were maintained within the Iberian Peninsula during the Pliocene, with a boost of species and lineage diversification during the Pleistocene, acting as one of the major glacial refugia for European taxa (Hewitt, 2000). However, its complex physiography and climatic heterogeneity have allowed the coexistence of species with diverse affinities (i.e., Eurosiberian and Mediterranean origins) and distinct evolutionary histories (Gómez & Lunt, 2007). For instance, mountain ranges helped to provide glacial refugia to many taxa and, thus, enabled population survival by allowing species to track suitable microclimates up or down altitudinal shifts (Gómez & Lunt, 2007; Hewitt, 1996). Due to its geographic and climatic characteristics, the Iberian Peninsula did not constitute a homogeneous and continuous refugium, but rather a fragmented area with multiple isolated refugia where speciation occurred followed by post‐glacial dispersal (Abellán & Svenning, 2014; Gómez & Lunt, 2007; Martínez‐Freiría et al., 2020).

Amphibians represent an excellent model to investigate the topographic and environmental drivers of spatial patterns of diversity at regional scales because of their generally poor dispersal abilities and reduced geographic ranges that make them more susceptible to the effect of scale on PD and PE relative to other tetrapods (Daru et al., 2020).

The main goal of this study is to evaluate if general drivers of spatial patterns of PD and PE at the global scale are also predictive of PD and PE at regional scales, using Iberian amphibians as a case study. Our objective is to map phylogenetic diversity and endemism, identify regional hotspots of diversity, and test whether water‐energy availability, topography, and historical climatic refugia can explain current spatial patterns of biodiversity. We hypothesize that topography along with contemporary climate and historical climate stability will be predictors of phylogenetic diversity and phylogenetic endemism, but the strength of these predictors may be weaker at the regional scale than they generally are at global scales.

## 2 MATERIALS AND METHODS

### 2.1 Species distribution data and phylogeny

The taxonomy of Iberian amphibians has been revised multiple times in the last decade mostly due to increased phylogeographic and morphological studies, while some cryptic species are still being unveiled using genomic data (Dufresnes et al., 2020). The taxonomy used in this study is the one accepted by the Spanish Herpetological Society (Carretero et al., 2018), which recognized 27 species (Table S1). Amphibian distributions in the IP, which are well documented in the Portuguese and Spanish atlases of amphibians and reptiles (Loureiro et al., 2008; Pleguezuelos et al., 2002), and the Spanish Herpetological Society database (http://siare.herpetologica.es/) were compiled and assembled in a 10 × 10 km grid (Carvalho et al., 2017) (Figure S1), which included 7955 grid cells and 51,541 occurrences.

We used an ultrametric interspecific phylogenetic tree, in which every tip represents each of the 27 Iberian species included in our study, previously inferred using Bayesian inference from an alignment of mitochondrial (12 S, 16 S and CYTB) and nuclear (RAG1) DNA sequences (Carvalho et al., 2017).

### 2.2 Diversity measures

Species distributions and the phylogenetic tree were used to map spatial patterns of diversity, which have been previously published (see Carvalho et al., 2017). For each 10 km grid cell, we estimated PD as the sum of branch lengths on the spanning path linking the occurring taxa to the root of the tree. To estimate PE for each cell, we divided the length of each branch by its geographical range and summed this value across all branches occurring in the cell (Rosauer et al., 2009).

In each cell, we computed PD and PE in R v 3.4.1 (R Core Team, 2017). We calculated PD using the *picante* package (Kembel et al., 2010) and PE, using “phylo.endemism” function (http://davidnipperess.blogspot.com.au/2012/07/phyloendemism‐r‐function‐for.html).

### 2.3 Null models

We used the “tip shuffle” null model (Cavender‐Bares et al., 2004) to test for a significant contribution of evolutionary relationships to phylogenetic diversity measures. Significantly higher PD values than expected by chance in a location provide a strong indication of the presence of anciently diverged lineages across the phylogeny, whereas significantly higher PE values provide a strong indication of the presence of phylogenetically distant lineages or of members of narrowly distributed clades.

The “tip shuffle” method randomized the phylogenetic relationships between species by shuffling the allocation of species to tree tips, while keeping constant the tree topology and the community data matrix. The shuffling was performed with 999 replicates and calculated expected PD (or PE), standardized effect size (SES), and *p*‐value in each grid cell for each randomization using the *picante* package in R.

### 2.4 Drivers of diversity patterns

We compiled spatial data on topographic and environmental variables related to the water‐energy availability, topography, and historical climatic instability hypotheses (Table 2 and Figure S2). All variables were standardized to match the 10 × 10 km resolution of species distribution data, using the *raster* (Hijmans, 2016) and *maptools* packages (Bivand & Lewin‐Koh, 2017) in R.

**TABLE 2:** Compiled predictors of diversity and corresponding source, initial resolution and temporal scope.

Type	Predictor	Source	Initial resolution	Temporal scope
Water‐Energy	Water vapor	Worldclim v.2.1	2.5′	1970–2000
	Rainfall	Worldclim v.2.1	2.5′	1970–2000
	Temperature	Worldclim v.2.1	2.5′	1970–2000
	Minimum normalized difference vegetation index (NDVI)	Global Land Cover Facility	8 km	1981–2006
	Maximum NDVI	Global Land Cover Facility	8 km	1981–2006
Topography	Altitude	SRTM data downloaded from Worldclim	30″	
	Slope			
	TPI			
	TRI			
Climatic instability	Mean Instability since LIG	Worldclim v.1.4	2.5′ and 30″	120 ky–present
	Mean, Sum and Standard Deviation (SD) Instability since LGM	PaleoView v.1.1	2.5′	21 ky–present

*Note*: Maximum NDVI values represent maximum productivity, whereas Minimum NDVI values are attributed more consistent cloud cover and water vapor effects since both reduce NDVI.

*Source*: WorldClim database (http://www.worldclim.org), Global Land Cover Facility (www.landcover.org), PaleoView version 1.1 (https://github.com/GlobalEcologyLab/PaleoView/releases).

We derived the remaining measures of habitat heterogeneity slope, Topographic Position Index (TPI) (Weiss, 2001), and Terrain Ruggedness Index (TRI) (Riley et al., 1999) from altitude, using the *raster* package in R.

We used climatic instability, i.e., climate fluctuations to represent cyclic fluctuations of the past, during the Quaternary. We define instability as the average euclidean distance between the scores of a principal component analysis (PCA) using the current climate and its projection using the same variables for a different period. Thus, higher values point toward higher instability and lower values toward stability. In each cell, we used PC1 and PC2 scores for each of the time periods considered and calculated the euclidean distance between each time and the subsequent one (as seen in Abellán & Svenning, 2014). We inferred two measures of historical climate instability (McDonald‐Spicer et al., 2019): instability since the Last Inter‐ Glacial (hereafter LIG) and since the Last Glacial Maximum (hereafter LGM). LIG was estimated using 19 bioclimatic variables (Table S2) from current conditions and from three historical periods: 6 ky, 21 ky, and 120 ky before the present (bp).

To estimate LGM, we repeated the previous procedure, but focused on a restricted set of bioclimatic variables (average precipitation, maximum temperature, and minimum temperature) and with a higher frequency of predictions (every 500 years). Furthermore, due to its higher frequency of climatic predictions, we calculated the mean, sum, and SD of the overall Euclidean distances. The historical bioclimatic data used to estimate both instability measures was predicted based on the Community Climate System Model (CCSM) simulation model which is the single model available in PaleoView (Fordham et al., 2017).

### 2.5 Hypotheses testing

We tested the ability of the water and energy availability, topography, and historical refugia (i.e., climate instability) hypotheses (see Table 1) to explain the observed spatial patterns of PD and PE (response variables).

We analyzed the pairwise Pearson correlation among variables and used the correlation matrix in a hierarchical clustering method (“*hclust*” in R) to group highly correlated variables and aid the selection of the final set (Table S3 and Figure S3). Additionally, we calculated the variance inflation factor (VIF) of the total set of variables using the *usdm* package (Naimi et al., 2014) in R. We then reduced multicollinearity by removing variables with VIF higher than 2.5 through a stepwise procedure. We retained the variables that passed both tests to the subsequent analyses.

We scaled all predictors and log‐transformed PE to meet model assumptions of normality. We also removed the species *Calotriton arnoldii* from our analysis as its distribution range is highly restricted and leads to exceptionally high PE scores in the 2 grid cells in which the species occurs, hampering model fit.

Furthermore, we tested for spatial independence of the model residuals by performing a spatial autocorrelation analysis. We estimated and assessed the significance of the Moran's *I* statistic at increasing spatial distances (0.52 decimal degrees increments) and visualized the respective correlogram (Figure S4). This analysis was conducted using the *pgirmess* package (Giraudoux, 2017) in R. As we found significant spatial autocorrelation in model residuals (*p*‐value < .05), for both linear regression models for PD and PE, we then used Spatial Autoregressive Models (SARs), which can address the spatial autocorrelation present in our data by incorporating a spatial weights matrix where the relationship between the residuals at each site and those at neighboring sites is defined.

To that purpose, we first identified the neighbors of each grid cell, using the *spdep* R package (Bivand et al., 2013; Bivand & Piras, 2015). For each model, we considered neighbors all grid cells up to the Euclidean distance wherein, according to our correlograms, there was no statistically significant autocorrelation, i.e., first nonsignificant value of the correlogram. Next, we derived a spatial weight matrix based on distances by weighting the neighbors with a standardized coding scheme (Kissling & Carl, 2008).

We tested all possible combinations of predictors with SARs, using a model selection framework implemented in the *spdep* R package. Additionally, as our null models describe the randomness of evolutionary relationships, we repeated spatial autocorrelation analysis (Figure S5) and SAR model selection using the mean expected PD and PE values from the null model alongside other predictors (as seen in Rosauer & Jetz, 2015) to account for the effect of random phylogenetic relationships and understand how the predictors explain the contribution to observed PD and PE:

PD∼PDnull+predictors

PE∼PEnull+predictors

All models were ranked according to Akaike Information Criterion (AIC) scores. For the best models, we also calculated variable significance (*p*‐value < .05) and goodness‐of‐fit through Nagelkerke pseudo *R* ^2^ (Nagelkerke, 1991) using the *spdep* R package.

## 3 RESULTS

### 3.1 Diversity measures

**FIGURE 1:**
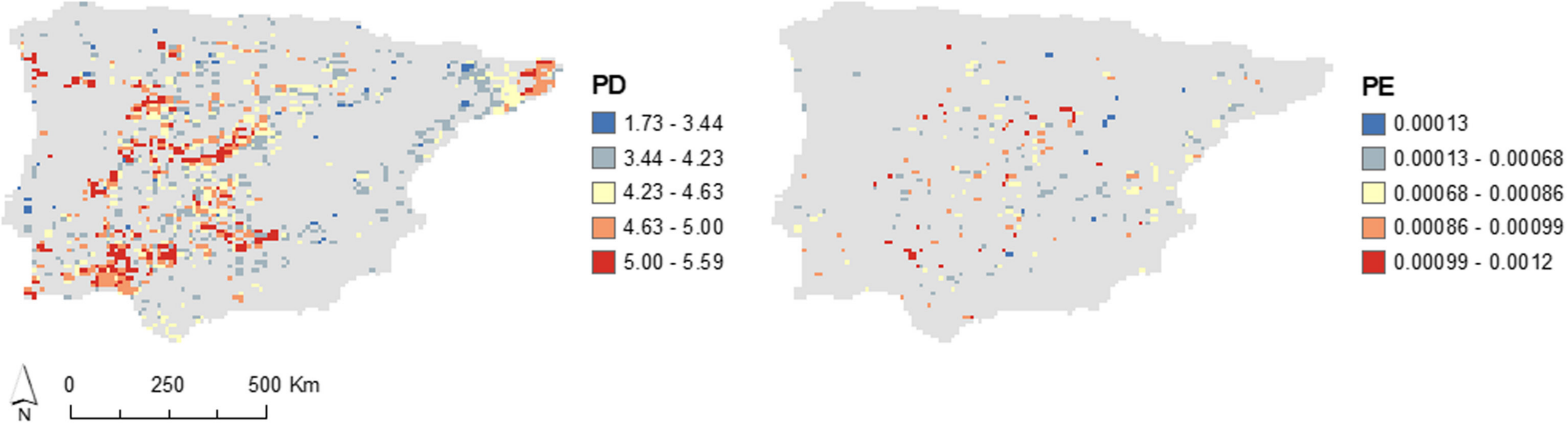
Spatial diversity patterns. Phylogenetic diversity and phylogenetic endemism values of cells where the observed value is significantly higher (*p*‐value > .95) than the null model.

Spatial patterns of PD and PE (Figure S6) seem to be unevenly distributed in the Iberian Peninsula. The results of the “tip shuffle” null model evidenced several locations where the observed PD and PE were significantly higher than expected (*p*‐value > .95) (Figure 1 and Figure S7). We note, however, that the range of PD and PE values which differed significantly from the null expectation, was wide, ranging from low to high values.

### 3.2 Drivers of diversity patterns

**FIGURE 2:**
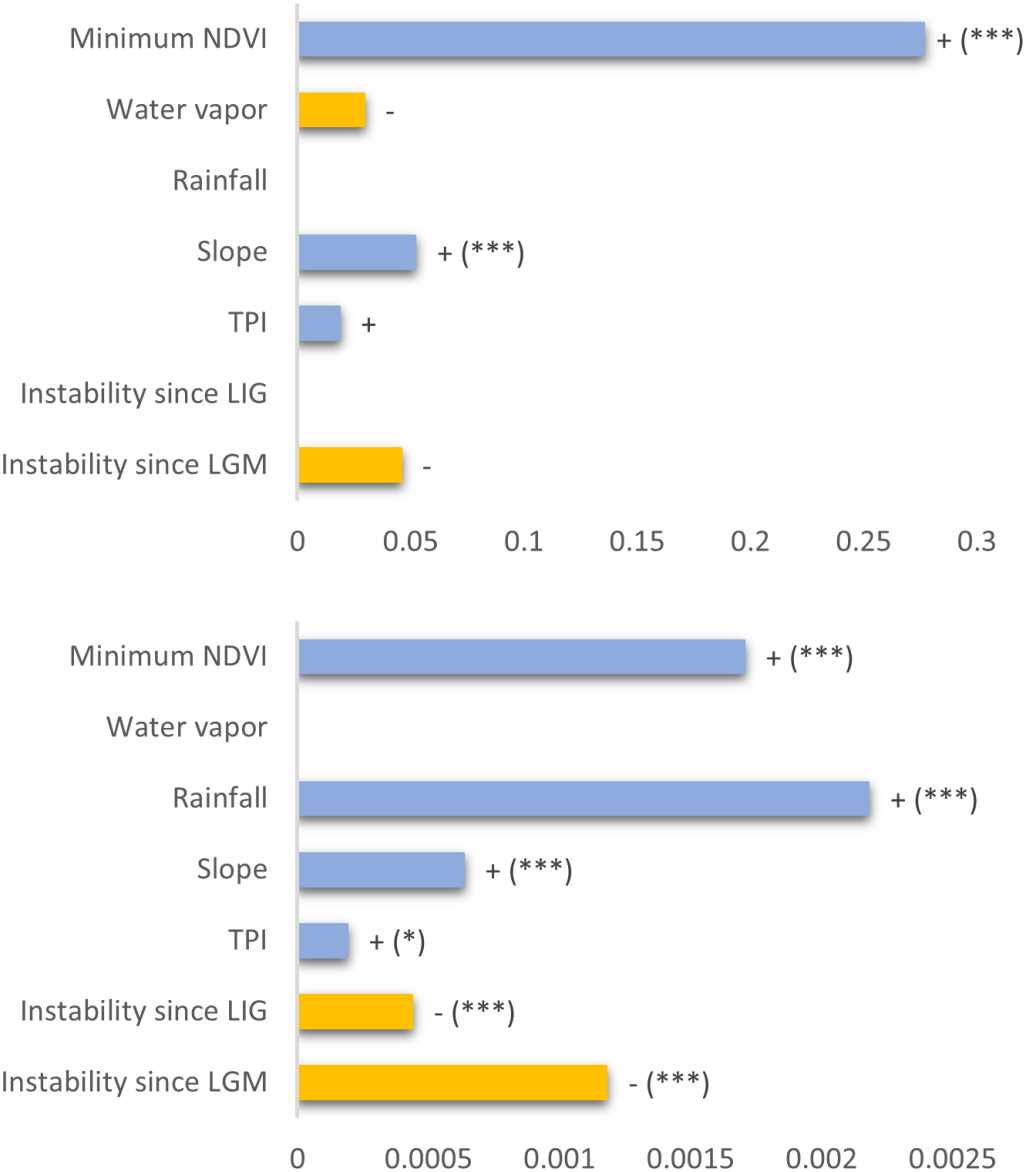
Coefficients from spatial autoregressive models used to assess the contribution of geographic and climatic predictors to (a) phylogenetic diversity (*PD ~ predictors*) and (b) phylogenetic endemism (*PE ~ predictors*) according to the best model. Blue bars (+) depict positive correlations, and yellow bars (−) depict negative correlations. Absent bars indicate predictors not included in the best model. Significance codes are: (‘***’) *p* < .001; (‘**’) *p* < .01; (‘*’) *p* < .05.

The coefficients of each variable used as a predictor of amphibian PD varied considerably. Based on the ΔAIC scores of our best five models, which were quite similar (Table S4), the best model (Nagelkerke pseudo *R* ^2^ = .21), included five predictors: minimum NDVI, water vapor, slope, TPI, and LGM. Energy availability, represented by minimum NDVI, was the most significant correlate of amphibian PD pattern. Slope, a measure of habitat heterogeneity, and TPI also had a significantly positive correlation with PD. In contrast, water vapor and LGM were not significant (Figure 2).

Regarding PE, the best SAR model (Nagelkerke pseudo *R* ^2^ = .31), based on ΔAIC scores (Table S5), included six predictors: minimum NDVI, rainfall, slope, TPI, LIG, and LGM. Current patterns of energy and water availability, represented by minimum NDVI and rainfall, were the most significant predictors of amphibian phylogenetic endemism patterns. Habitat heterogeneity, represented by slope and TPI, were also significantly correlated with PE. Additionally, the two measures of climatic instability (LIG and LGM) had a negative significant correlation with PE; therefore, higher PE was found in locations that remained climatically stable through time.

**FIGURE 3:**
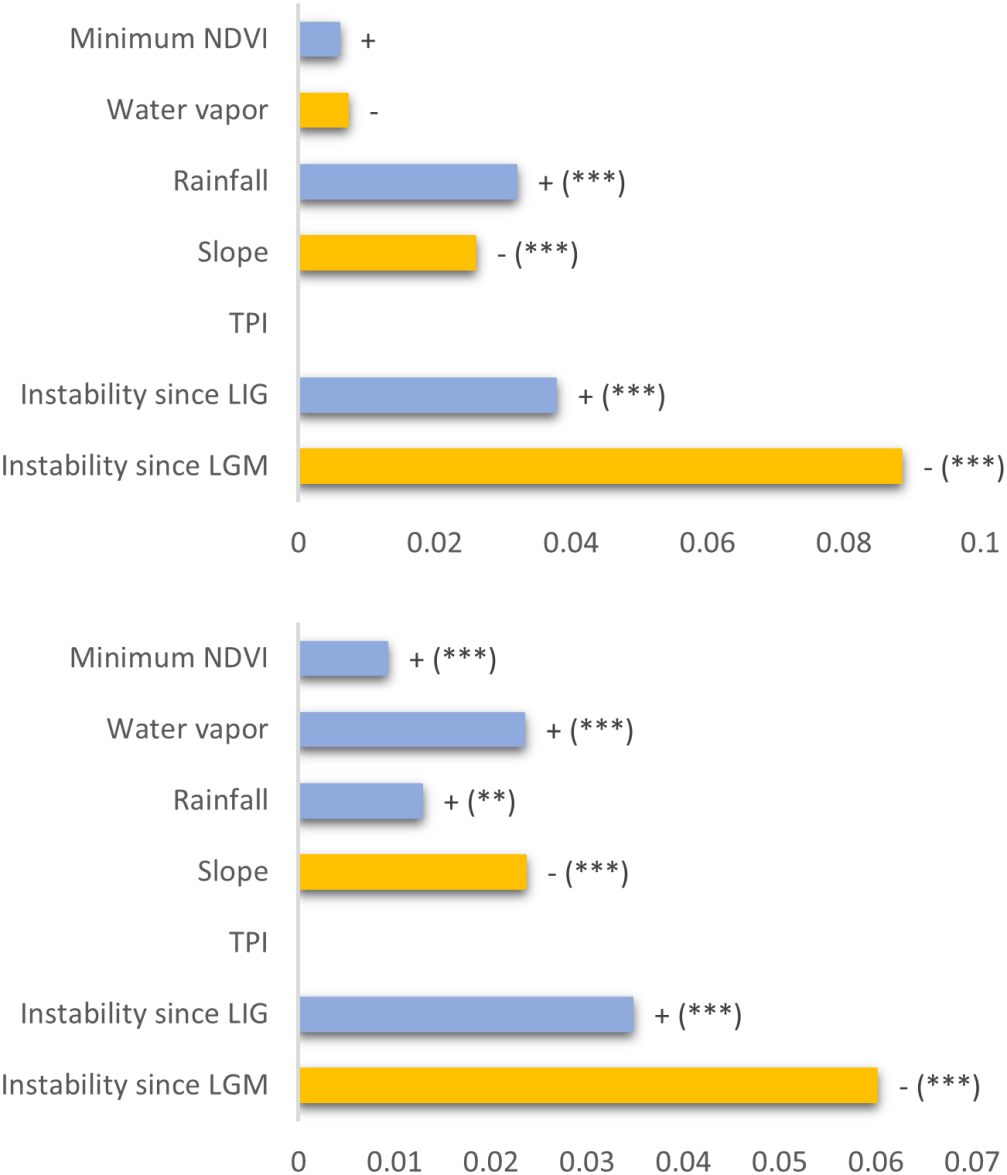
Coefficients from spatial autoregressive models used to assess the contribution of geographic and climatic predictors to (a) phylogenetic diversity using PD expected from the null model randomizing evolutionary relationships as a predictor (*PD ~ PD null + predictors*) and (b) phylogenetic endemism using PE expected from the null model randomizing evolutionary relationships as a predictor (*PE ~ PE null + predictors*). Coefficients are presented according to the best model. Blue bars (+) depict positive correlations to PD, and yellow bars (−) depict negative correlations. Absent bars indicate predictors not included in the best model. Significance codes are: (‘***’) *p* < .001; (‘**’) *p* < .01; (‘*’) *p* < .05.

When using expected PD based on the “tip shuffle” null model as a predictor to account for the effect of species richness, the best model (Nagelkerke pseudo *R* ^2^ = .93), based on ΔAIC scores (Table S6), explaining observed PD comprised six predictors: minimum NDVI, water vapor, rainfall, slope, LIG, and LGM. LGM was the most significant predictor of PD. Furthermore, PD had a significant positive correlation with rainfall and LIG and a significant negative correlation with slope (Figure 3). Minimum NDVI and water vapor, despite being included in the model, did not show a significant correlation with PD.

The best model (Nagelkerke pseudo *R* ^2^ = .93), based on ΔAIC scores (Table S7), explaining observed PE when using expected PE based on the “tip shuffle” null model as a predictor included six predictors: minimum NDVI, water vapor, rainfall, slope, LIG, and LGM. Phylogenetic endemism was significantly correlated with minimum NDVI, both predictors of water availability and LIG. Slope and LGM had a significantly negative correlation with spatial patterns of PE, with LGM being the most significant driver.

## 4 DISCUSSION

### 4.1 Drivers of diversity patterns

Our study presents an analysis of possible drivers of amphibian PD and PE in the Iberian Peninsula. We tested three hypotheses regarding topographic and environmental predictors of amphibian diversity and, according to our results, water‐energy and historical climatic instability are the most important drivers of amphibian diversity in Iberia.

Our null “tip shuffle” models show that although the majority of sites did not evidence a significant deviation from the null model, some locations do demonstrate that the phylogenetic structure differs significantly from this expectation, i.e., observed PD and PE were significantly higher than expected. These results were congruent with the “independent swap” null model for PE (see Figure S8), where species occurrences were reassigned to random grid cells without replacement while keeping constant the species frequency of occurrence (i.e., the total number of grid cells where each species occurs) and species richness in each grid cell. However, deviations did not seem to have a clear regional pattern, i.e., significant sites were few and quite scattered across the study area. Furthermore, as both “tip shuffle” null models were calculated in the same manner, when comparing PD and PE results, we can infer that locations where only significant PE values are found might be attributed to the community of species present, which may have an overall smaller range.

Overall, our results demonstrate that energy (i.e., vegetation) and water availability were significant predictors in our models. Since amphibians, through their life cycle, depend on both terrestrial and aquatic habitats, high accessibility to water is needed for survival. Therefore, both water availability and productive energy may allow co‐existence of more individuals and maintenance of larger, more viable populations, which is essential to lower extinction risk and recovering from disturbances. Furthermore, both predictors can be important range‐limiting factors as amphibians are cold blooded and highly dependent on environmental temperature and humidity.

When accounting for the effect of the number of species, historical climatic instability is the most significant predictor of diversity. LGM is negatively correlated with PD and PE, which is consistent with the historical refugia hypothesis. Indeed, many studies indicate that the range of some Iberian amphibian species contracted into climatic refugia during unsuitable periods and expanded after climatic amelioration (Gómez & Lunt, 2007; Hewitt, 2000). Such was the case, e.g., of *Chioglossa lusitanica* (Alexandrino et al., 2000), *R. temporaria* (Dufresnes et al., 2020), *R. iberica* (Teixeira et al., 2018), *Ichthyosaura alpestris* (Recuero et al., 2014), *Lissotriton boscai* (Teixeira et al., 2015), *Pelobates cultripes* (Gutiérrez‐Rodríguez et al., 2017a), *Pleureodeles waltl* (Gutiérrez‐Rodríguez et al., 2017b), *Alytes cisternasii* (Gonçalves et al., 2009), and *Salamandra salamandra* (Antunes et al., 2018) However, contrary to our expectations, when accounting for the effect of the number of species, LIG was positively related to PD and PE, which contradicts our historical refugia hypothesis. The lack of consistent results from our climatic instability measures could be attributed to the fluctuation of climate‐stable areas throughout time, meaning that there could have been a change or lack of consistency in the areas that have experienced stable climates throughout time. It is also possible that LIG might not be an accurate representation of climatic instability over time due to longer averages and huge gaps in which spatial patterns may have varied differently, i.e., we might be underestimating climatic variability occurring during periods in which we do not have available data. Given these results, we believe instability might be a better predictor of intraspecific (i.e., phylogeographic) diversity, as verified in other regional studies (Barratt et al., 2017; Carnaval et al., 2009), since it is a relatively recent event and lineages have not had enough time to diverge into separate species.

Most studies focusing on identifying drivers of PD and PE have been undertaken on a continental scale (e.g. Araújo et al., 2008; Murali et al., 2021; Rosauer & Jetz, 2015). However, these metrics rely on the composition of local communities, and the assembly processes differ from continental to regional scales (e.g. Münkemüller et al., 2014; Wiens & Donoghue, 2004) because biotic and abiotic filters often operate at different spatial scales (Webb et al., 2002). For example, the continental climate might initially condition species with similar climate affinities to coexist at the regional scale. Since species with similar traits tend to be more phylogenetic‐related, niche conservatism tends to support the hypothesis of community assembly at regional scales. However, at local scales, other factors such as microhabitat filtering, competition (Sommer et al., 2017), and recent biogeographic history may conduct two phylogenetically related species to avoid coexistence (phylogenetic overdispersion) (Algar et al., 2011). Therefore, we believe that the lower goodness of fit of our models could be partially explained not only by the high stochasticity of our system but also by the fact that drivers of diversity may differ across different spatial scales. Hence, despite being significant, the strength of the tested predictors in our case study is weaker than it tends to be at global scales.

The neutral theory suggests that individuals in a community, irrespective of species, share equivalent prospects of reproduction and death. However, recent studies (Chave, 2004; Clark, 2012; Leigh, 2007) have criticized this theory. Given all the necessary parameters to measure and calibrate this theory, it is quite difficult to test it with empirical data (Gotelli & McGill, 2006). Additionally, simulation studies have demonstrated that phylogenetic structure is weakly explained by the neutral theory (Missa et al., 2016). Alternatively, null models offer much better statistical power and are easier to use with empirical data (Gotelli & McGill, 2006). As such, we have opted to use a null model instead of a neutral model. However, we do note the similarity of our null model results is consistent with results obtained with simulated data under both neutral species assemblage and under the habitat filtering hypothesis (Miller et al., 2017).

Patterns of biodiversity can only be explained by complex models with many interacting predictors, our hypothesized drivers of amphibian diversity focused on present‐day factors (climate and topography) and historical climate until 120 kyr BP while the great majority of amphibian diversification occurred during the earlier stages of Cenozoic (66 myr BP) (Roelants et al., 2007; Wiens & Donoghue, 2004). Although our hypothesized drivers might represent climate fluctuations at a larger scale, each period has its particularities that might influence the biogeographical history of the species. Additionally, due to lack of appropriate data, ancient historical climatic fluctuations related to plate tectonics and orogeny, which are likely important predictors to explain amphibian diversity patterns in Iberia, were not accounted for in our study.

Lastly, we note that PE calculations are scale dependent (Daru et al., 2020), thus, by studying drivers of diversity on a regional scale, we are disregarding that spatially restricted species within the Iberian Peninsula could be globally widespread (e.g., *Rana temporaria, Rana dalmatina, Ichthyosaura alpestris*—restricted in Iberia but widespread in Europe). Additionally, the presence of species with large geographical ranges relative to the study area could also have weakened our ability to detect a relationship with the tested predictors. The inclusion of dispersal barriers might be important to improve our ability to detect relationships with major climate and environmental drivers. However, its inclusion in our study was not possible due to the lack of accurate data.

## 5 CONCLUSIONS

Understanding which topographic and environmental factors drive patterns of PD and PE, at both global and local scales, is highly important as they have been shown to be effective surrogates for adaptive and neutral variation in conservation planning (Hanson et al., 2017; Ponce‐Reyes et al., 2014). Thus, developing our knowledge on these matters is exceptionally important, as it could be used for the conservation of locations where the use of such diversity measures is not possible due to a lack of phylogenetic data.

Our study highlights that water‐energy availability and historical climate instability are the most significant predictors of the patterns of diversity observed in Iberian amphibians. However, as predicted, the strength of these predictors in our case study is weaker than it tends to be at global scales. Thus, additional drivers should be investigated, and we recommend caution when interpreting these predictors as surrogates for different components of diversity and advocate the assessment of spatial diversity patterns for conservation planning whenever possible.

## AUTHOR CONTRIBUTIONS

**Dan Rosauer:** Conceptualization (equal); formal analysis (supporting); investigation (supporting); methodology (supporting); software (supporting); supervision (equal); validation (equal); writing – review and editing (supporting). **Pedro Tarroso:** Formal analysis (supporting); investigation (supporting); writing – review and editing (supporting). **Guillermo Velo‐Antón:** Formal analysis (supporting); investigation (supporting); writing – review and editing (supporting). **Silvia B Carvalho:** Conceptualization (equal); data curation (supporting); funding acquisition (lead); methodology (lead); project administration (lead); resources (lead); supervision (equal); validation (equal); writing – review and editing (lead). **Maria João Paúl:** Data curation (lead); formal analysis (lead); investigation (lead); software (lead); visualization (lead); writing – original draft (lead).

## FUNDING INFORMATION

Project NGC—Next Generation Conservation is funded by FEDER funds through the Operational Program for Competitiveness Factors—COMPETE and by National Funds through FCT—Foundation for Science and Technology (PTDC/BIA‐BIC/3545/2014). MJP was funded by a PhD Grant through Fundação para a Ciência e Tecnologia (SFRH/BD/08664/2020). PT was funded by Fundação para a Ciência e Tecnologia (CEECIND/01464/2017). GVA was supported by Fundação para a Ciência e Tecnologia (CEECIND/00937/2018) and recently by a Ramón y Cajal research grant (Ref. RYC‐2019‐026959‐I/AEI/10.13039/50110 0011033). SBC was funded by an individual scientific employment program contract through Fundação para a Ciência e Tecnologia (CEECIND/01464/2017). Work co‐funded by the project NORTE‐01‐0246‐FEDER‐000063, supported by Norte Portugal Regional Operational Programme (NORTE2020), under the PORTUGAL 2020 Partnership Agreement, through the European Regional Development Fund (ERDF).

## Supporting information


Appendix S1

## Data Availability

All raw data used in this study is openly available in public repositories, except the phylogenies which are available upon request.
