# Supplementary material for: Environmental and topographic drivers of amphibian phylogenetic diversity and endemism in the Iberian Peninsula
Source: Ecol Evol. 2023 Jan 6;13(1):e9666. doi: 10.1002/ece3.9666 (PMC9817204; doi:10.1002/ece3.9666)
Supplement: Supplementary file 1 — Appendix S1 [file ECE3-13-e9666-s001.pdf]

Supporting information

S1 Table. List of Iberian amphibian species (Taxa), their conservation status and distribution range. Conservation status is represented according to International Union for Conservation of Nature and Natural Resources (IUCN) Red List (http://www.iucnredlist.org/): Least Concern (LC), Near Threatened (NT), Vulnerable (VU), Endangered (EN), Critically Endangered (CR), Not Evaluated (NE). Species range is characterized as: Endemic (range restricted to the Iberian Peninsula), European restricted (restricted range within Europe), European Widespread (wide range within Europe), Mediterranean (range around the Mediterranean basin) and Palearctic (wide range through Europe and extending to Asia or northern Africa or the Arabian Peninsula).

**Taxa**	**Conservation status**	**Species Range**
Alytes cisternasii (Boscá, 1879)	NT	Endemic
Alytes dickhilleni (Arntzen & García-París, 1995)	VU	Endemic
Alytes obstetricans (Laurenti, 1768)	LC	European restricted
Bufo calamita[1] (Laurenti, 1768)	LC	European widespread
Bufo spinosus (Daudin, 1803)	NE	Endemic
Calotriton arnoldi (Carranza & Amat, 2005)	CR	Endemic
Calotriton asper (Dugès, 1852)	NT	European restricted
Chioglossa lusitanica (Bocage, 1864)	VU	Endemic

Discoglossus pictus (Otth, 1837)	LC	Recently introduced
Discoglossus galganoi (Capula, Nascetti, Lanza, Bullini & Crespo, 1985)	LC	Endemic
Hyla meridionalis (Boettger, 1874)	LC	Mediterranean
Hyla molleri (Bedriaga, 1890)	NE	Endemic
Ichthyosaura alpestris (Laurenti, 1768)	LC	European widespread
Lissotriton boscai (Lataste, 1879)	LC	Endemic
Lissotriton helveticus (Razoumowsky, 1789)	LC	European widespread
Pelobates cultripes (Cuvier, 1829)	NT	European restricted
Pelodytes ibericus (Sánchez-Herráiz, Barbadillo, Machordom & Sanchiz, 2000)	LC	Endemic
Pelodytes punctatus (Daudin, 1802)	LC	European restricted
Pelophylax perezi (Seoane, 1885)	LC	European restricted
Pleurodeles waltl (Michahelles, 1830)	NT	Mediterranean
Rana dalmatina (Bonaparte, 1840)	LC	Palearctic
Rana iberica (Boulenger, 1879)	NT	Endemic

Rana pyrenaica (Serra-Cobo, 1993)	EN	European restricted
Rana temporaria (Linnaeus, 1758)	LC	Palearctic
Salamandra salamandra (Linnaeus, 1758)	LC	European widespread
Triturus marmoratus (Latreille, 1800)	LC	European restricted
Triturus pygmaeus (Wolterstorff, 1905)	NT	Endemic

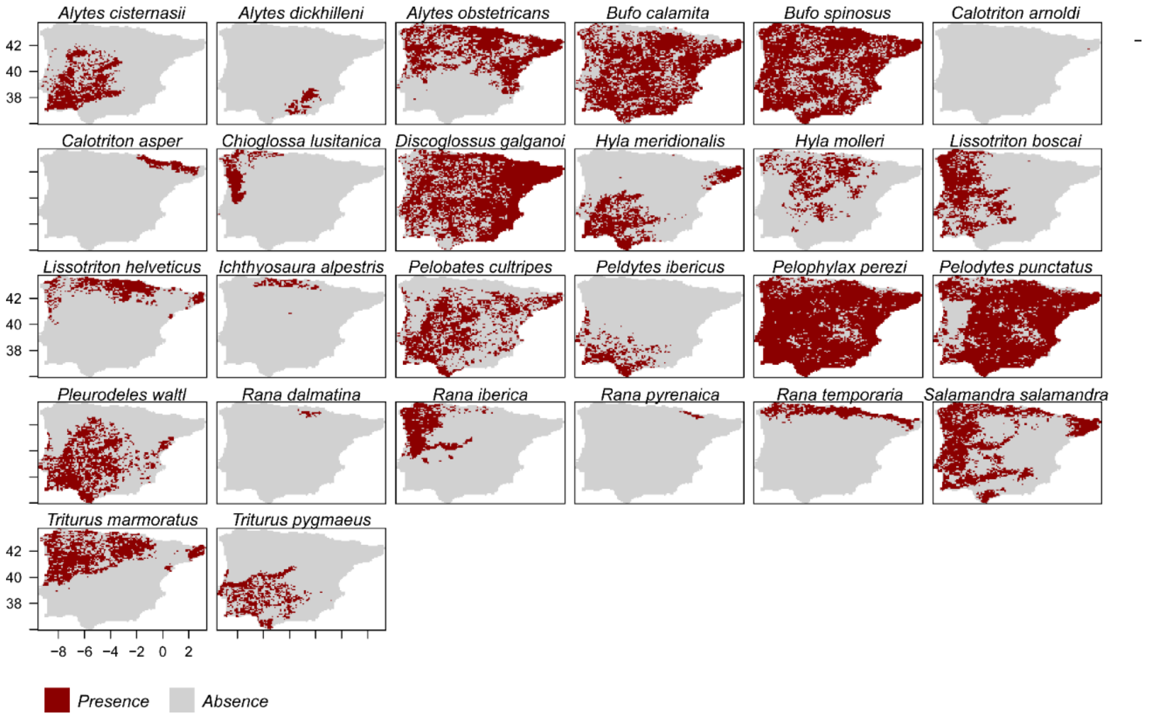

S1 Fig. Spatial distribution of amphibian species in the Iberian Peninsula.

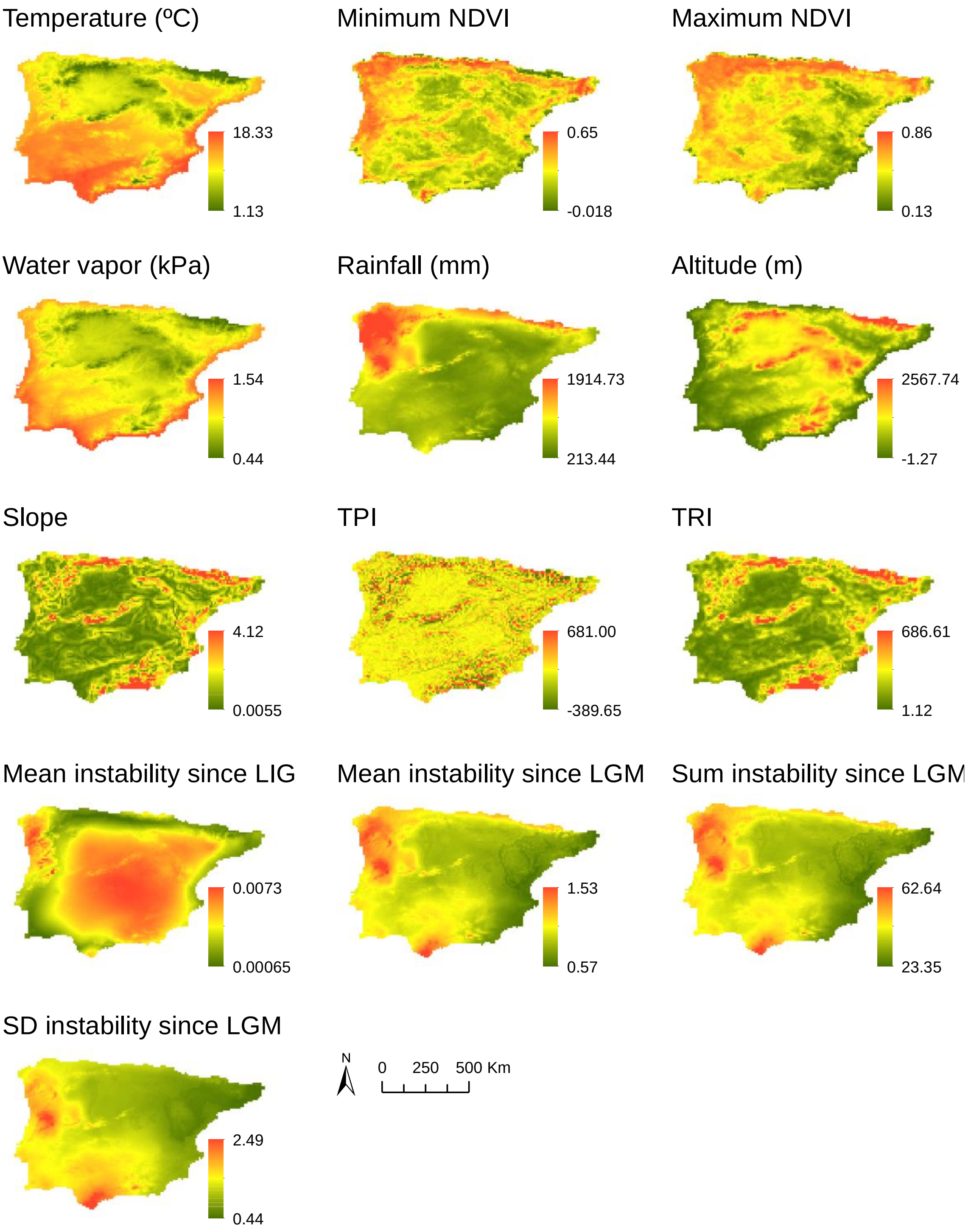

S2 Fig. Compiled predictors of diversity and their corresponding range and unit.

S2 Table. Bioclimatic variables used to estimate instability since LIG. Source: WorldClim database (http://www.worldclim.org/).

Code	Description
BIO1	Annual Mean Temperature
BIO2	Mean Diurnal Range (Mean of monthly (max temp - min temp))
BIO3	Isothermality (BIO2/BIO7) (* 100)
BIO4	Temperature Seasonality (standard deviation *100)
BIO5	Maximum Temperature of Warmest Month
BIO6	Minimum Temperature of Coldest Month
BIO7	Temperature Annual Range (BIO5-BIO6)
BIO8	Mean Temperature of Wettest Quarter
BIO9	Mean Temperature of Driest Quarter
BIO10	Mean Temperature of Warmest Quarter
BIO11	Mean Temperature of Coldest Quarter
BIO12	Annual Precipitation
BIO13	Precipitation of Wettest Month
BIO14	Precipitation of Driest Month
BIO15	Precipitation Seasonality (Coefficient of Variation)
BIO16	Precipitation of Wettest Quarter
BIO17	Precipitation of Driest Quarter
BIO18	Precipitation of Warmest Quarter
BIO19	Precipitation of Coldest Quarter

S3 Table. Pairwise Pearson correlation among variables. Retained variables for subsequent analyses are in bold.

	Altitude	Max NDVI	**Min NDVI**	**Rainfall**	**Slope**	Temperature	**TPI**	TRI	**Water vapor**	Mean instability since LGM	**SD instability since LGM**	**Instability since LIG**	Sum instability since LGM
Altitude	1.00												
Max NDVI	0.06	1.00											
**Min NDVI**	-0.12	0.64	1.00										
**Rainfall**	0.04	0.63	0.52	1.00									
**Slope**	0.32	0.06	0.13	0.18	1.00								
Temperature	-0.86	-0.33	-0.12	-0.35	-0.33	1.00							
**TPI**	0.30	0.04	0.01	0.12	0.02	-0.28	1.00						
TRI	0.41	0.08	0.13	0.23	0.91	-0.41	0.17	1.00					
**Water vapor**	-0.94	-0.12	0.06	-0.07	-0.21	0.88	-0.24	-0.28	1.00				
Mean instability since LGM	-0.13	0.48	0.33	0.76	-0.01	0.02	0.10	0.04	0.17	1.00			

**SD instability since LGM**	-0.29	0.21	0.15	0.40	-0.12	0.38	0.07	-0.09	0.40	0.86	1.00		
**Instability since LIG**	0.20	-0.41	-0.30	-0.40	-0.22	0.09	-0.02	-0.22	-0.26	-0.17	0.00	1.00	
Sum instability since LGM	-0.13	0.48	0.33	0.76	-0.01	0.02	0.10	0.04	0.17	1.00	0.86	-0.17	1.00

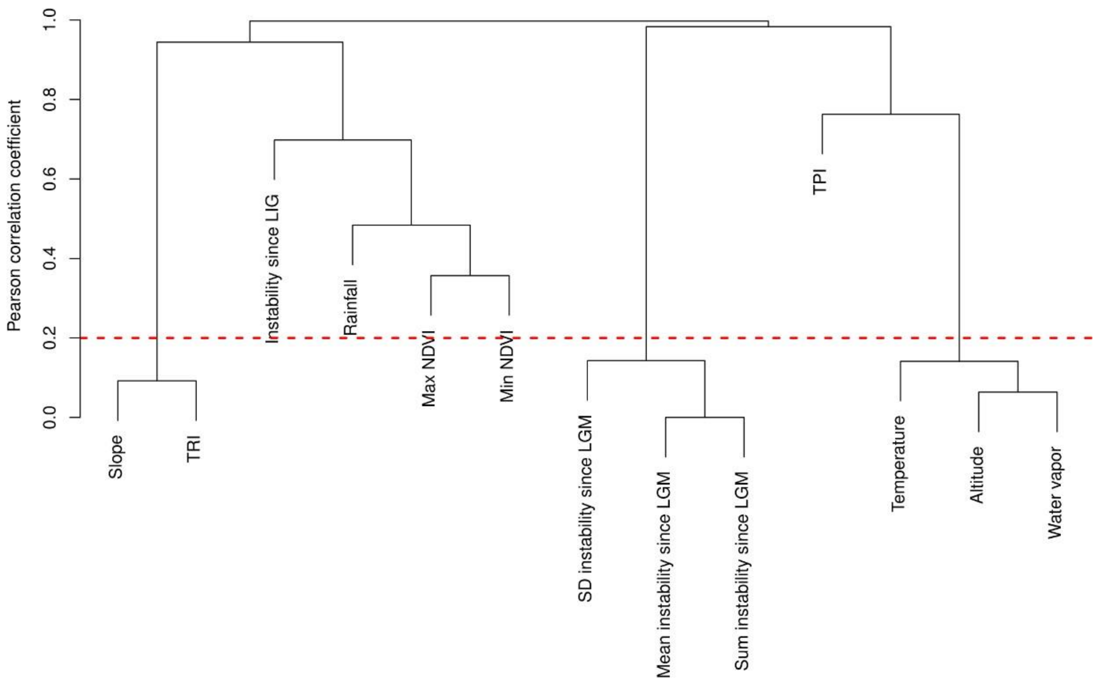

S3 Fig. Cluster dendrogram of hypothesized drivers of diversity based on the Pearson correlation coefficient, where lower distances represent higher correlation.

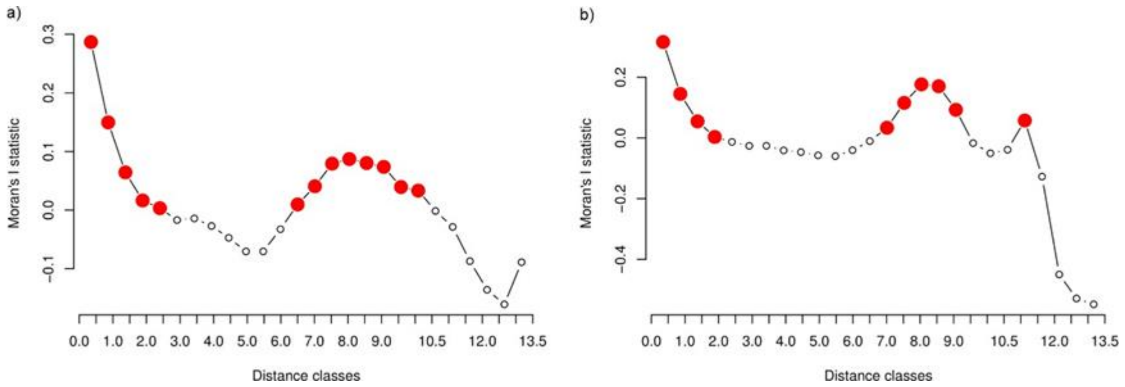

S4 Fig. Correlograms representing Moran's I statistic at increasing spatial distances, where (a) PD ~ predictors, (b) PE ~ predictors. Statistically significant Moran's I values are plotted in red.

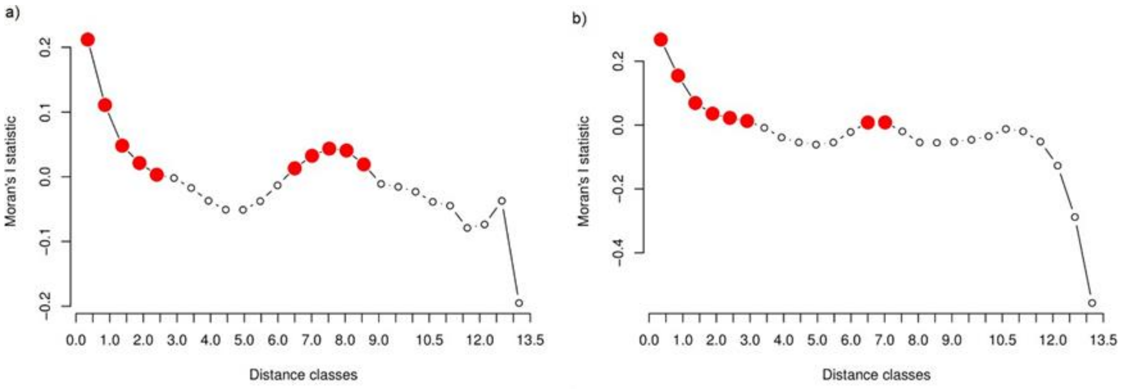

S5 Fig. Correlograms representing Moran's I statistic at increasing spatial distances, where (a) PD ~ PD null 'tip shuffle' + predictors and (b) PE ~ PE null 'tip shuffle' + predictors. Statistically significant Moran's I values are plotted in red.

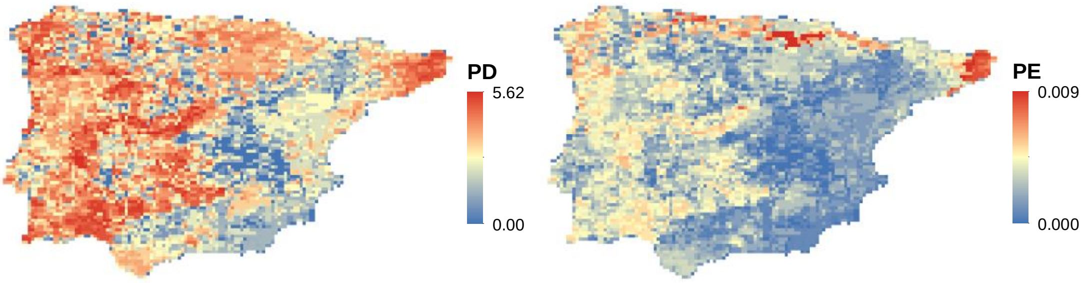

S6 Fig. Spatial diversity patterns (PD and PE) of amphibian species in the Iberian Peninsula.

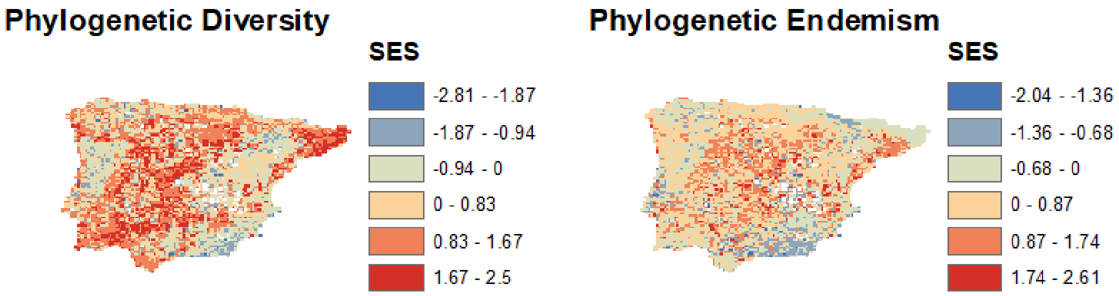

S7 Fig. Standardized effect size (SES) of the null model.

S4 Table. Coefficients of best PD models (PD ~ predictors), AIC scores calculated relative to the best model (ΔAIC = 0) and model weight.

Best model is in bold.

Minimum NDVI	Water vapor	Rainfall	Slope	TPI	Instability since LIG	Instability since LGM	ΔAIC	Model weight
**0.28**	**-0.030**	**0**	**0.052**	**0.019**	**0**	**-0.046**	**0**	**0.094**
0.28	-0.038	0	0.051	0	0	-0.041	0.23	0.084
0.28	-0.032	0.035	0.048	0	0	-0.067	0.67	0.067
0.28	-0.025	0.032	0.049	0.017	0	-0.069	0.75	0.064
0.28	0	0.042	0.051	0.023	0	-0.077	0.83	0.062

S5 Table. Coefficients of best PE models (PE ~ predictors), AIC scores calculated relative to the best model (ΔAIC = 0) and model weight.

Best model is in bold.

Minimum NDVI	Water vapor	Rainfall	Slope	TPI	Instability since LIG	Instability since LGM	ΔAIC	Model weight
**0.0017**	**0**	**0.0022**	**0.00063**	**0.00019**	**-0.00044**	**-0.0012**	**0**	**0.50**
0.0017	-0.00012	0.0021	0.00061	0.00017	-0.00048	-0.0011	1.14	0.28
0.0017	-0.00020	0.0021	0.00059	0	-0.00050	-0.0011	2.87	0.12
0.0017	0	0.0023	0.00062	0	-0.00043	-0.0012	3.37	0.092
0.0017	0	0.0024	0.00069	0.00018	0	-0.0013	8.95	0.0057

S6 Table. Coefficients of best PD models using PD expected from the null model randomizing evolutionary relationships as a predictor (PD ~ PD null 'tip shuffle' + predictors), AIC scores calculated relative to the best model (ΔAIC = 0; highlighted in bold) and model weight.

Minimum NDVI	Water vapor	Rainfall	Slope	TPI	Instability since LIG	Instability since LGM	ΔAIC	Model weight
**0.0060**	**-0.0073**	**0.032**	**-0.026**	**0**	**0.038**	**-0.088**	**0**	**0.19**
0	0	0.039	-0.025	0	0.040	-0.092	0.019	0.19
0.0053	0	0.036	-0.025	0	0.040	-0.092	0.24	0.17
0	-0.0064	0.035	-0.025	0	0.038	-0.089	0.26	0.16
0.0060	-0.0082	0.032	-0.026	-0.0023	0.037	-0.088	1.56	0.086

S7 Table. Coefficients of best PE models using PE expected from the null model randomizing evolutionary relationships as a predictor (PE ~ PE null 'tip shuffle' + predictors), AIC scores calculated relative to the best model (ΔAIC = 0; highlighted in bold) and model weight.

Minimum NDVI	Water vapor	Rainfall	Slope	TPI	Instability since LIG	Instability since LGM	ΔAIC	Model weight
**0.0092**	**0.023**	**0.013**	**-0.024**	**0**	**0.035**	**-0.060**	**0**	**0.68**
0.0092	0.024	0.013	-0.023	0.0011	0.035	-0.060	1.70	0.29
0.011	0.020	0	-0.023	0	0.032	-0.051	7.60	0.015
0.011	0.021	0	-0.023	0.0013	0.032	-0.051	9.18	0.0070
0	0.025	0.017	-0.023	0	0.035	-0.061	15.17	0.00035

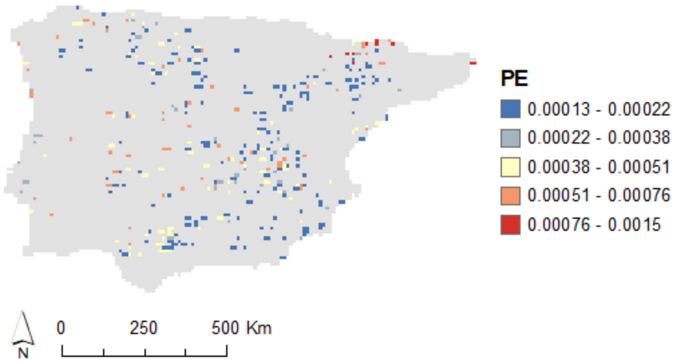

S8 Fig. Spatial diversity patterns. PE values of cells where the observed value is significantly higher (p-value > 0.95) than the 'independent swap' null model.
